# Supplementary material for: Perish and publish: Dynamics of biomedical publications by deceased authors
Source: PLoS One. 2022 Sep 14;17(9):e0273783. doi: 10.1371/journal.pone.0273783 (PMC9473445; doi:10.1371/journal.pone.0273783)
Supplement: S1 File — (PDF) [file pone.0273783.s002.pdf]

Supporting Information

The rate and extent of publications in particular journals by deceased authors prior to their deaths could influence the rate of publications after those authors' deaths. Among the 38907 publications by deceased authors that contain journal titles in the PubMed record, 63 journals that had at least 10 publications either before or after death were examined for correlation. The Spearman's test did not show any meaningful correlation, however, between the rates of prior death publications with deceased publications, although Pearson's test showed mild correlation, which was probably driven by the one journal that is the top-most frequent in both before and after death publications (S1 Fig).

We next examined whether the number of prior before death publications in a journal had any influence on the number of post-death publications by these authors. We restricted our analysis to the top 17 journals that published the most deceased author papers. An analysis of the raw numbers of these publications indicated that in general the number of prior to death publications by deceased authors in a journal did not particularly influence the number of papers published by them after death (S2A Fig). The raw numbers clearly indicated that some journals published markedly more papers from deceased authors than other journals in particular *PLOS One* and *Scientific Reports* (S2B Fig). However, this does not take into account the large numbers of papers that are published annually in these journals. We therefore scaled the raw number of publications to the number of overall papers published in 2019 for each of these 17 top journals. Once the scaled results were depicted, the frequency of deceased author publications from journals changed markedly, with *PLOS One* and *Scientific Reports* making a much smaller contribution to these deceased publications and other journals such as *Nature Genetics* now increasingly apparent (S2B Fig). The scaled frequency of deceased author publications in the journals also appeared to be more influenced by the frequency of prior-to-death publications by the same authors (S2B Fig). We measured the extent of the association between prior and post-death publications in a given journal, by estimating the odds of publishing a deceased author publication in a journal by the extent of before-death publications in that journal. The total publications for each journal in the year 2019 were used for the calculation of odds. The odds-ratio was calculated using Fisher's exact test with contingency tables specific for each journal. The p-value for each journal was adjusted by the Benjamini & Hochberg method. This analysis surprisingly revealed that for *Scientific Reports* ($p=0.04$) a deceased author had a higher likelihood of publishing in this journal after death than before (S2C Fig). We examined whether deceased author publications were published in journals with lower impact factors than papers published by those same authors prior to their deaths. We plotted the distribution of publications by the top 6 most prolific deceased authors ranked by the 5-year impact factor of these journals as of 2019, over an 11-year time span, from 5 years prior to and 5 years post death of these authors. For those journals where a 5-year impact factor was not available we used the most recent 2-year impact factor or set it to zero if no impact factor was available. The results indicated that overall, there were no major differences in the apparent perceived quality of publications by deceased authors comparing their papers from 5 years prior to 5 years post their deaths, using journal impact factors as a surrogate of quality (S3 Fig). More uncertainty in the results are apparent however as the time span increases after the year of death with the relatively smaller number of publications at 4 and 5 years post death causing larger fluctuations in the range of impact factors (S3 Fig).

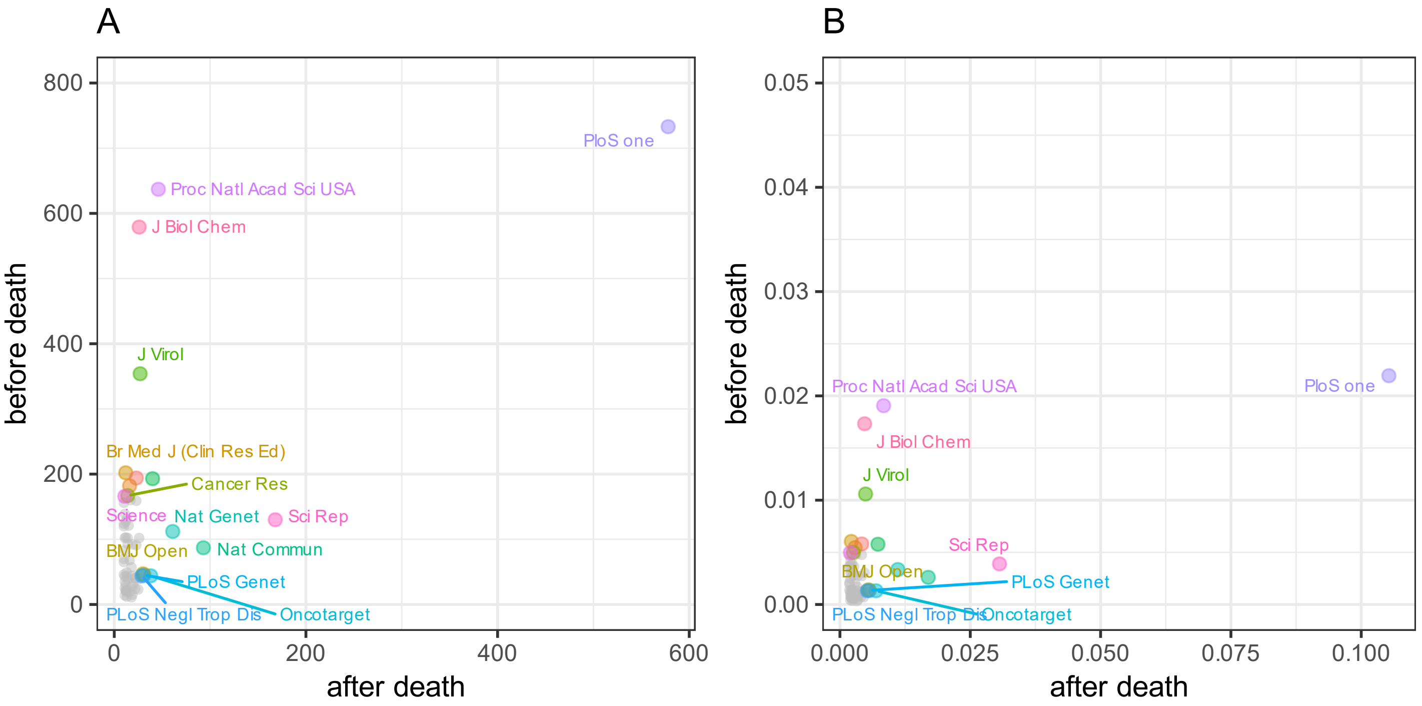

S1 Fig. Distribution of publications with deceased authors by Journal.
The top journals are depicted with the number of publications (A) and by the fraction (B). The before death publications depict the total number of publications in that journal by the total pool of deceased authors prior to their death and similarly for after death publications. The fraction is calculated separately for before- and after-death publications. Journals that published the most deceased author papers (17 journals) are shown in various colours.

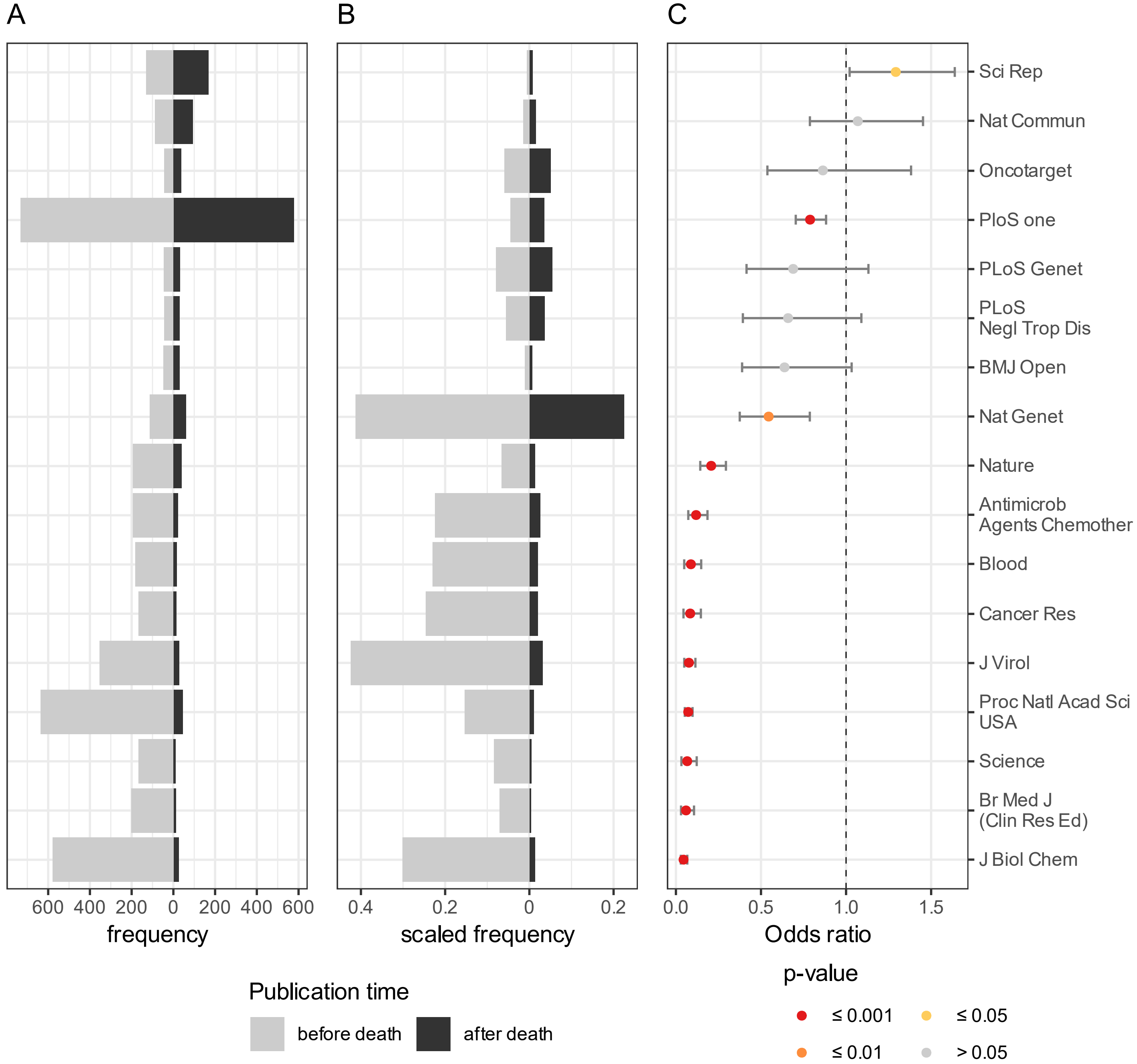

S2 Fig. Comparison between top 17 journals with deceased author publications.
A total of 17 journals are shown in the order of the number of deceased publications. (A) Raw number of publications before or after death. (B) The relative frequency of publications has been scaled to the total number of publications in the journal in the year 2019. Before death publications are in grey and post-death publications in black. (C) Odds ratios or measures of association of odds of a deceased author publishing in a journal based on before death publications in that journal. The total publications for each journal in the year 2019 were used for the calculation of odds. The odds-ratio was calculated using Fisher's exact test with contingency tables specific for each journal. The p-value for each journal in the plot was adjusted by the Benjamini & Hochberg method.

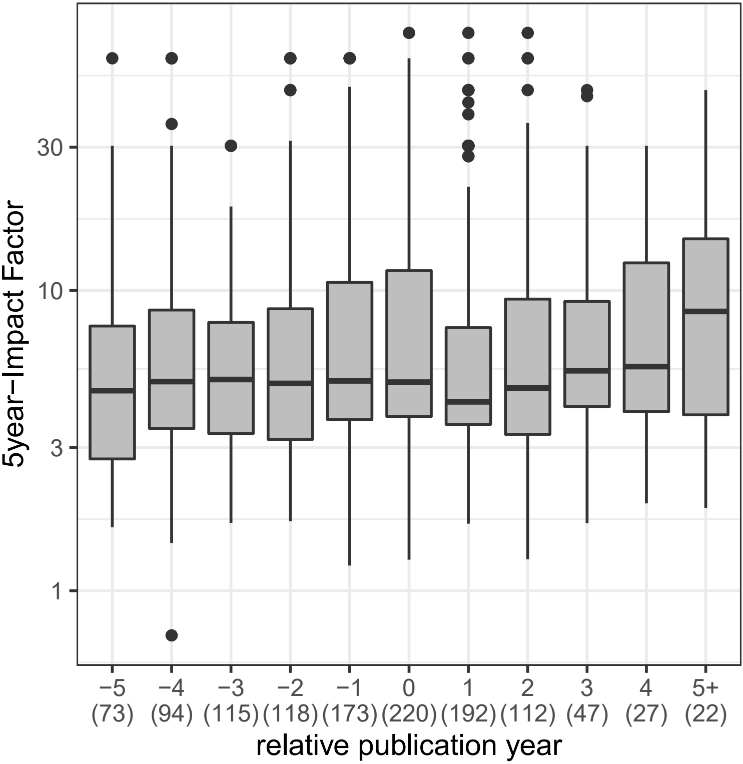

S3 Fig. Distribution of publications by most prolific deceased authors (n=6) ranked by impact factor of the journals around the year of death. The 5-year citation impact factors, as of 2019, were used. For the journals that did not have the 5-year impact factor in 2019, the most recent 2-year impact factor was used. If no impact factor was available, the impact factor was set to zero. Dark line is the median and box the interquartile range. Numbers in the parenthesis denote the number of papers.
