# Supplementary material for: Perish and publish: Dynamics of biomedical publications by deceased authors
Source: PLoS One. 2022 Sep 14;17(9):e0273783. doi: 10.1371/journal.pone.0273783 (PMC9473445; doi:10.1371/journal.pone.0273783)
Supplement: Attachment — Submitted filename: rebuttal letter PONE-D-21-35578.docx [file pone.0273783.s003.docx]

PONE-D-21-35578

Perish and Publish: Dynamics of Biomedical Publications by Deceased Authors

PLOS ONE

Rebuttal Letter

Dear Dr Jelte M. Wicherts
Academic Editor
PLOS ONE

Thankyou for your detailed comments on our manuscript.

We have carefully addressed each of the points raised by yourself and by Reviewer #2 and have listed our responses to each of the comments (*in italics*) below.

We note that Reviewer #1 made no recommendations that needed to be addressed.

Apart from one comment, we have complied with each of the requested changes to the manuscript and hope it now meets your requirements for acceptance.

*Editor:*

*First, Reviewer 1 rightly indicated that you present a substantial number of supplementary results and I agree that many of these results in later sections of the manuscript are less relevant, and could be placed in an (online) appendix. So a major revision would shorten the paper to present the main results but not the supplementary results with respect to journals.*

We have taken this recommendation on board and have now shortened the paper by removing the results concerning journal to the Supplementary information section, this has removed three Figures (former Figs 6, 7, 8; now S1 Fig, S2 Fig and S3 Fig) and shortened the number of Figs from 9 to 6.

*Reviewer #2:*

*Introduction:
- The current introduction is quite superficial. Authors need much better contact with the available literature about posthumous authorship.*

*- References 1-8 discuss a range of different issues and citing all of them after a generic claim is 1) a cop out, 2) an example of citation padding.
- References 11-15 too, discuss a range of different issues and citing all of them in one cluster is 1) a cop out, 2) an example of citation padding.*

We have added more text to the introduction and now clearly separated refences that cite issues relating broadly to authorship rights and responsibilities from those papers specifically related to the issues around deceased authors. *- When relying on the ICMJE definition, please make sure that the definition of authorship is accurately reflected. The ICMJE definition does NOT require "intellectual" contributions, it requires "substantial contributions". Also, when referring to the ICMJ, please avoid citing secondary sources that interpret the definition.*

We have added a specific section to the introduction citing the ICMJE criteria explicitly, and have removed the term re ‘intellectual’ contributions with ‘substantial’ as requested.

*Editor:*

*Second, I also agree with the second reviewer that you performed many exploratory tests to document particular patterns but that these appeared not to be based on any prior hypotheses. It is important to clearly identify tests that were data driven from the key tests about which you had prior hypotheses and to identify the former types of tests as exploratory and in need of fresh data to ensure replicability and robustness.*

*Reviewer #2*

*Results:
- There's a good bit of repetition in this section.
- I suggest that you use sub-headings to improve readability.*

As recommended we have added sub-headings to the Results section as requested.

*- Authors note that some journals have a policy regarding posthumous authorship. This should be added to the introduction with some examples.*

We have shortened the text and reduced repetition by removing the text around the previous Figs 6-8 and moved these to the Supplementary information and have added a sentence to the introduction stating updated journal policies re posthumous authorship.

*- In most of the provided analyses, a hypothesis is missing. Using "we wondered" is not necessarily helping because some could interpret this as an example of HARKING.*

We have removed the term ‘wondered’ and specifically stated the hypothesis that is being tested from the data now throughout the results section.

*Authors use a range of different statistical methods without clarifying the link between their hypothesis and used method.*

We have modified the wording in the results section to clarify which statistical test is being used in each scenario and what hypothesis it is being used to test for.

*Editor:*

*Third, you need to devote more attention to the main limitations of the database and data collection and be open and critical on what they might mean. Keep in mind that we at PLOS ONE value rigor over "newsworthiness" of the findings and so I urge you to be openly and meticulously discuss methodological issues such as those mentioned by the second reviewer. Here, I also noted the dramatic increase from null occurrences before 1999 and afterwards that look to me like they could be driven by changes in text section coverage or other factors that would be good to consider (e.g., by checking trends in funding statements as a control).*

We agree with this comment and have added a paragraph to the Discussion commenting on the limitations of our database and made a particular reference to the limitations on not finding any deceased author publications prior to 1999, including a reference to the limitations of only searching in the Acknowledgement sections of papers.

*Reviewer #2*

*Discussion:
- Please make sure that all results are included in the results section and methods are in the methodology. Currently some of the results and methods are in the discussion section.*

We have made reference to our results in the Discussion section but in all cases these results were already listed in the Results section (or now in the Supplementary information section) and hence we do not feel this observation is valid, as we did not discuss any results that were not already listed in the Results section.

*- References 20-27, 1) a cop out, 2) an example of citation padding.*

We have shortened the number of references listed here.

*Editor:*

*Fourth, the second reviewer raises specific issues related to the ICMJE definition and improper motives to include a deceased author and other issues that you need to carefully consider when revising your work.*

We have added a paragraph to include this point as a potential motive by living authors as to why they include deceased authors in publications.

We have uploaded a marked version of the manuscript with all changes in red text and also a new unmarked version of the revised manuscript as requested.

with many thanks

Christopher Hovens

Department of Surgery

University of Melbourne

Royal Melbourne Hospital

chovens@unimelb.edu.au
