# Supplementary material for: Perish and publish: Dynamics of biomedical publications by deceased authors
Source: PLoS One. 2022 Sep 14;17(9):e0273783. doi: 10.1371/journal.pone.0273783 (PMC9473445; doi:10.1371/journal.pone.0273783)
Supplement: Attachment — Submitted filename: Response to Reviwers PONE-D-21-35578R1.docx [file pone.0273783.s004.docx]

PONE-D-21-35578R1

Perish and Publish: Dynamics of Biomedical Publications by Deceased Authors

PLOS ONE

Rebuttal Letter

Dear Dr Jelte M. Wicherts
Academic Editor
PLOS ONE

Thankyou for your detailed comments on our manuscript.

We have carefully addressed each of the points raised by yourself and by Reviewer #2 and have listed our responses to each of the comments (*in italics*) below.

We have complied with each of the requested changes to the manuscript and hope it now meets your requirements for acceptance.

*Editor:*

*I agree with the reviewer that the text needs revision and a restructuring such that the methods are part of the main text rather than put at the end. The reviewer also makes specific points concerning the text, some inaccuracies, methodological issues, and earlier work in this area that you can readily address. I also noted that the text needs some further editing (note that PLOS ONE does not offer text editing to lower the APC) and another check of minor inaccuracies and ambiguities in the text.*

We have taken these recommendations on board and have revised and restructured the paper by moving the Materials and methods straight after the Introduction.

We have also addressed each of the issues raised by reviewer #2 (please see below).

I should also point out that we will make the database we have generated for this project available (reviewer #2 has incorrectly stated that we won’t) and in fact are attaching this database as a supplementary table S1 with this revised submission.

However, you should consider whether you want identifying information of the 1439 deceased authors in this dataset as currently it is present. This is because it needs to be, if anyone wants to reproduce our findings in full.

*Reviewer #2:*

*Please remove citation nr. 5, when using direct quotations, you cannot cite two independent sources!*

We have removed citation #5 as requested. *Authors note “For example, if an author passes away between formal acceptance of a peer-reviewed paper and its publication, there may be robust documentation of them meeting all four criteria.”, which negates the opening sentence of the exact same paragraph: “if these criteria were followed strictly it would be impossible for any deceased author to fulfill all four criteria.”*

This is a good point, as we agree the text didn’t make sense here.

We have changed the wording and added extra text to make this clearer and improve the logic here.

The new text is in lines 60-65.

*Authors note “Some biomedical journals, such as, BMJ Journals, Pediatric Anesthesia have now developed their own policies with respect to deceased authors, potentially fragmenting the consensus understanding of what constitutes authorship.”, I suggest authors to provide further explanation on some details of these policies and a clear explanation about why these policies fragment the consensus understanding.*

We agree with this comment and have now added new text clearly outlining the new policies of the *BMJ Journals* and of *Pediatric Anesthesia.*

This is outlined in the new text at lines 65-75 in the Introduction.

*Furthermore, creating a “consensus understanding” is vague: “consensus among who?”
Also, what is meant by consensus understanding? If, as authors claim in their opening statement, “The concept and definitions of what constitutes authorship in scientific publications has been much commented in the literature and numerous frameworks…”, there is no consensus about authorship to begin with! Creating a consensus about any authorship issue is almost impossible, so this is s flimsy rational anyway. I’d suggest using “consistent practice in the community”, “harmonized practice” or something similar so that you don’t imply that creating consensus is actually possible.*

To improve ambiguity around the understanding of what a consensus framework means we have removed the offending lines discussing the consensus framework from the Introduction. *A lot of what is currently mentioned in the results section especially what is under “Compiling a database of posthumous authors” should be in the “Methodology” section, which is currently after the acknowledgement section! PLease make sure that the paper has a solid structure and flow. At the moment, the paper meanders a good bit.*

We have removed the section ‘Compiling a database of posthumous authors’ from the Results section and this is now in the Materials and Methods. As requested, the Materials and Methods has now been placed before the Results section.

*A lot of what is currently mentioned in the results section especially what is under “The cumulative change in deceased author publications over time” should be mentiond in the Intro, as the rationale for the study.*

We have added extra text at the end of the Introduction as to the rationale and aims of the study taken from the Results section *“The cumulative change in deceased author publications over time”* as requested*.* This is new text lines 77-81.

*Furthermore, another rationale could be that adding dead authors increases the average number of authors per publication, which has been reported to exacerbate all ethical issues of authorship at some level (*[*https://link.springer.com/article/10.1007/s11948-021-00352-3*](https://link.springer.com/article/10.1007/s11948-021-00352-3)*)*

We have added new text in the Discussion lines 314-316 addressing this issue and quoting the refence supplied. *The sentence “It is possible that some cases of posthumous authorship in our analysis include the use of a high profile researchers profile to improve a study’s impact” is badly written*

We have rewritten this sentence to improve clarity new lines 312-314 in the Discussion. *Refernce #26, Kosmulski 2021 has some really useful content for this paper. I suggest authors to carefully read and add some recommendations to their own paper. For instance, authors could highlight the fact that "Neither HTLM not PDF versions on journals' Web pages inform that one of the authors was deceased" and suggest that publishers should be more proactive and transparent in declaring whether the author was dead at the time of submission, during the review process, between acceptance and publication etc.*

This is a good point and we have added new text in the Discussion lines 324-332, highlighting many of the points addressed by Kosmulski 2021, including our own recommendations as to how to potentially resolve the issue of the status of deceased authors in the literature.

We have uploaded a marked version of the manuscript with all changes in red text and also a new unmarked version of the revised manuscript as requested.

with many thanks

Christopher Hovens

Department of Surgery

University of Melbourne

Royal Melbourne Hospital

chovens@unimelb.edu.au
